# Exercise Capacity in Children and Adolescents With Congenital Heart Disease: A Systematic Review and Meta-Analysis

**DOI:** 10.3389/fcvm.2022.874700

**Published:** 2022-05-04

**Authors:** Yenny Villaseca-Rojas, Javiera Varela-Melo, Rodrigo Torres-Castro, Luis Vasconcello-Castillo, Guillermo Mazzucco, Jordi Vilaró, Isabel Blanco

**Affiliations:** ^1^Programa de Magíster en Fisiología Clínica del Ejercicio, Facultad de Ciencias, Universidad Mayor, Santiago, Chile; ^2^Servicio de Kinesiología, Unidad de Medicina Física y Rehabilitación, Hospital Clínico UC-CHRISTUS, Santiago, Chile; ^3^Department of Physical Therapy, Faculty of Medicine, University of Chile, Santiago, Chile; ^4^International Physiotherapy Research Network (PhysioEvidence), Barcelona, Spain; ^5^Institut d'Investigacions Biomèdiques August Pi i Sunyer (IDIBAPS), Barcelona, Spain; ^6^Instituto Cardiovascular de Rosario, Rosario, Argentina; ^7^Universidad del Gran Rosario, Rosario, Argentina; ^8^Blanquerna School of Health Sciences, Global Research on Wellbeing (GRoW), Universitat Ramon Llull, Barcelona, Spain; ^9^Department of Pulmonary Medicine, Hospital Clínic, University of Barcelona, Barcelona, Spain; ^10^Biomedical Research Networking Center on Respiratory Diseases (CIBERES), Madrid, Spain

**Keywords:** pediatrics, heart defects, congenital malformations, cardiopulmonary exercise test, oxygen consumption, six-minute walking test

## Abstract

**Background:**

Congenital heart disease (CHD) entails structural defects in the morphogenesis of the heart or its main vessels. Analyzing exercise capacity of children and adolescents with CHD is important to improve their functional condition and quality of life, since it can allow timely intervention on poor prognostic factors associated with higher risk of morbidity and mortality.

**Objective:**

To describe exercise capacity in children and adolescents with CHD compared with healthy controls.

**Methods:**

A systematic review was carried out. Randomized clinical trials and observational studies were included assessing exercise capacity through direct and indirect methods in children and adolescents between 5 and 17 years-old. A sensitive analysis was performed including studies with CHD repaired participants. Additionally, it was sub-analyzed by age range (< and ≥ 12 years old). Two independent reviewers analyzed the studies, extracted the data, and assessed the quality of the evidence.

**Results:**

5619 articles were found and 21 were considered for the review. Eighteen articles used the direct exercise capacity measurement method by cardiopulmonary exercise test (CPET). The CHD group showed significant differences in peak oxygen consumption (VO_2_peak) with a value of −7.9 ml/Kg/min (95% CI: −9.9, −5.9, *p* = 0.00001), maximum workload (Wmax) −41.5 (95% CI: −57.9, −25.1 watts, *p* = 0.00001), ventilatory equivalent (VE/VCO_2_*) slope* 2.6 (95% CI: 0.3, 4.8), oxygen pulse (O_2_ pulse)−2.4 ml/beat (95% CI: −3.7, −1.1, *p* = 0.0003), and maximum heart rate (HRmax) −15 bpm (95% CI: −18, −12 bpm, *p* = 0.00001), compared with healthy controls. Adolescents (≥ 12 yrs) with CHD had a greater reduction in VO_2_peak (−10.0 ml/Kg/min (95% CI: −12.0, −5.3), *p* < 0.00001), Wmax (−45.5 watts (95% CI: −54.4, −36.7), *p* < 0.00001) and HRmax (−21 bpm (95% CI: −28, −14), *p*<0.00001).

**Conclusion:**

Suffering CHD in childhood and adolescence is associated with lower exercise capacity as shown by worse VO_2_peak, Wmax, VE/VCO_2_ slope, O_2_ pulse, and HRmax compared with matched healthy controls. The reduction in exercise capacity was greater in adolescents.

**Systematic Review Registration:**

www.crd.york.ac.uk/prospero/display_record.php?RecordID=208963, identifier: CRD42020208963.

## What is Known

Exercise capacity is one of the main factors that affect health-related quality of life, prognosis, risk of morbidity, and early mortality from cardiovascular, metabolic, or respiratory disease.

Analyzing exercise capacity in children and adolescents with CHD is important to improve their functional condition and quality of life, since it can allow timely intervention on poor prognostic factors associated with higher risk of morbidity and mortality.

## What the Study Adds

VO_2_peak, HRmax, Wmax, and O_2_ pulse were significantly lower in children and adolescents with partially or fully repaired CHD compared with healthy controls.

## Introduction

Congenital heart disease (CHD) entails structural defects in the morphogenesis of the heart or its main vessels (1). They are the most common congenital defect in children worldwide, with an average prevalence of 8.22 per 1000 live newly born, ranging from 2.4 to 13.7 (2, 3). According to intracardiac morphology and physiology, they are classified as acyanotic and cyanotic, and according to its severity as either simple, or complex (1).

For its treatment, there are both corrective and palliative surgeries (1). Due to medical advances, greater and better diagnostic, surgical and postoperative care technology, it is expected that more than 90% of children with CHD will currently survive to adulthood (4). However, despite the increase in the life expectancy of children with CHD, the residual defects that may remain after surgery can have a negative effect on both morbidity and mortality (5).

It has been reported that physical capacity in children with CHD is lower compared to healthy controls with limited exercise capacity and a shorter lifespan related to health (6). Limited exercise capacity favors a more sedentary lifestyle, a situation that can be maintained into adulthood (7). Accordingly, less physical activity increases the risk of overweight and obesity in children with CHD, which means an additional health burden (8).

Exercise capacity is one of the main factors when assessing health-related quality of life, prognosis, risk of morbidity, and early mortality from cardiovascular, metabolic, or respiratory disease (9). It can be evaluated by a standardized laboratory test such as cardiopulmonary exercise test (CPET) or standardized field tests such as the six-minute walking test (6MWT), shuttle walking test (SWT), time up and go (TUG), or similar tests (10). The CPET, which assesses the maximum oxygen consumption (VO_2_max) or peak (VO_2_peak) and measures ventilatory efficiency, has obtained a prognostic value in adults with acquired heart failure and CHD, by identifying subjects with limited cardiovascular reserve (11). Studies that consider the measurement of VO_2_peak in subjects with cyanotic CHD and palliative surgery for complex CHD highlighted it as an independent predictor of death or hospitalization due to a cardiovascular event (12, 13). In children with chronic diseases, VO_2_peak can predict adverse or unfavorable outcomes (6). The ventilatory equivalent for carbon dioxide production (V_E_/VCO_2_) has been shown to have high sensitivity as a predictor of mortality in subjects with various CHD (14).

Analyzing exercise capacity of children and adolescents with CHD is important to improve their functional condition and quality of life, since it can allow timely intervention on poor prognostic factors associated with higher risk of morbidity and mortality.

This review aims to systematically analyse studies to summarize exercise capacity, assessed with laboratory tests or standardized field tests, in children and adolescents with CHD compared with their healthy counterparts, using a meta-analysis of observational studies. Our purposes are to find out if the main exercise capacity variables such as VO_2_peak or Wmax are reduced in children and adolescents with CHD, and to find out if the cardiac response to exercise is also influenced.

## Methods

### Protocol and Registration

We performed a systematic review using Preferred Reporting Items for Systematic Reviews and Meta-analyses (PRISMA) guidelines (15). The review was registered in the International Prospective Register of Systematic Reviews (PROSPERO) CRD42020208963.

### Criteria for Considering the Studies in This Review

We included randomized clinical trials (RCTs) or observational studies (cross-sectional, longitudinal, case-control, and cohort) in children and adolescents with a diagnosis of CHD. The included studies aimed to determine the physical capacity in patients with CHD. Additionally, the studies should report VO_2_peak, maximal workload (Wmax), distance walked in the 6MWT (6MWD), or similar measurements obtained from objective tests. All editorials, letters, conference publications, review articles, systematic reviews, meta-analyses, *in vivo* and *in vitro* studies were excluded.

### Search Strategies and Data Resources

We reviewed seven databases: Embase, Cochrane Central Register of Controlled Trials (CENTRAL), CINAHL, Web of Science, PubMed/MEDLINE, Scopus, and SciELO, from their inception to 01 June, 2021 and conducted manual searches using the followings terms: a) [(Congenital heart disease) OR (Congenital heart defects) OR (Congenital heart surgery) OR (Fontan operation) OR (Fontan circulation) OR (Fontan patient) OR (Fontan physiology) OR (Fontan procedure) OR (Tetralogy of Fallot) OR (Interventricular communication) OR (Atrial communication)] AND [(Exercise capacity) OR (Physical capacity) OR (Exercise tolerance) OR (Cardiopulmonary exercise test) OR (CPET) OR (Walking test) OR (Walk test) OR (6MWT) OR (Shuttle walking test) OR (SWT) OR (distance walked) OR (Oxygen uptake) OR (Oxygen consumption) OR (Wingate Anaerobic Test) OR (Timed Up and Go) OR (TUG) OR (Exercise Test) OR (sit to stand) OR (step test) OR (STS)] AND [(Children) OR (Adolescents) OR (Pediatrics) OR (Childhood) OR (Pediatric)].

The selected terms were combined using Boolean logical operators (OR, AND, NOT). Moreover, we performed a manual search of the references that were included in the selected articles. All the references were analyzed in Rayyan software, a web-based tool (16).

### Reviewing Procedure and Study Selection

The review was performed independently by two investigators (YVR-JVM), who independently reviewed all articles titles and abstracts identified in the search strategy. The full text of potentially eligible studies was then read to verify their suitability for final inclusion. All studies that did not fulfill the predefined criteria were excluded, and their bibliographic details were listed with the specific reason for exclusion.

### Data Extraction and Methodological Quality Assessment

Two investigators (YVR-JVM) independently extracted data from the selected articles and recorded them in an *ad hoc* spreadsheet of relevant data. This included author, country, year of publication, sample size, study design, age of subjects, diagnoses, evaluation instruments, evaluated variables, and results. Data from the first assessment were considered for randomized or non-randomized clinical trials. Differences obtained from data extraction were resolved by consensus. In the case of not reaching an agreement, a third investigator (RTC) resolved the differences. If some relevant data were not in the article, the author was contacted to request the information.

Assessment of the methodological quality of the primary articles was carried out using the quality assessment tools from the National Heart, Lung, and Blood Institute (NHLBI) (17). Each tool contains criteria based on which internal validity and risk of bias are evaluated. The criteria are evaluated as “yes” “no” or “other” (not reported, not applicable, or not determinable), and an overall rating is provided for each study based on the items rated with an affirmative answer (> 75% = good, 50–75% = fair, < 50% = poor).

### Data Synthesis and Analysis

We reported summaries of the association between the outcomes for each study in terms of mean differences using Review Manager 5 (RevMan, Copenhagen: The Nordic Cochrane Center, The Cochrane Collaboration, 2014). We obtained combined measurements of effect for each primary outcome through meta-analysis under a random-effect model, due to the expected heterogeneity between the studies. Statistical heterogeneity was measured through the *I*^2^ statistic and classified as low (*I*^2^ <25%), moderate (*I*^2^ 25–50%) or high (*I*^2^ >50%). A sensitivity analysis was conducted including studies with only children and adolescents surgically repaired. Additionally, when possible, a subanalysis by age group (< and ≥ 12 years) was performed in the group of patients with surgical correction.

## Results

### Study Selection

**Figure 1:**
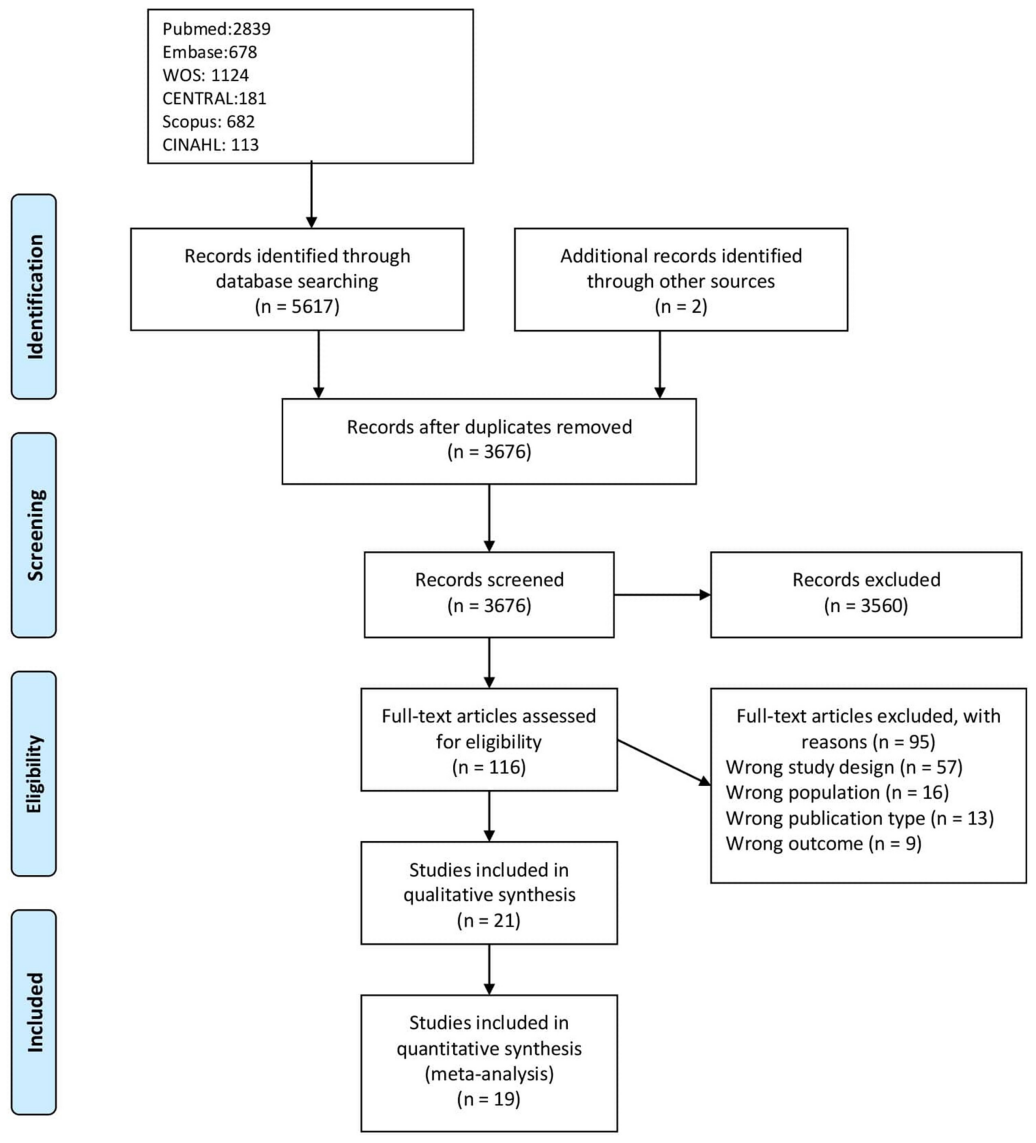
PRISMA flowchart of the studies.

The flow chart of the study selection process is shown in Figure 1. In the initial search of the selected databases, 5619 potential studies were identified. Of the 107 studies assessed as full text, we excluded 57 for wrong study designs, 16 for wrong population, 13 for wrong publication type, and nine due to a wrong outcome. In total, 21 studies fulfilled the criteria for eligibility and were included in the review.

### Characteristics of the Included Studies

Fifteen studies were conducted in Europe (18–32), three in the United States (33–35), one in Brazil (36), one in Japan (37), and one was conducted in Egypt (38). Regarding the design, 20 were cross-sectional (18–23, 25–38), and only one was an RCT (24). The characteristics of the included studies are summarized in Table 1.

**Table 1:** Characteristics of the included studies.

**Authors, year**	**Country**	**Group**	**Participants diagnosis**	**Severity/Repaired**	**Participants n (Female/Male)**	**Age** **(years)**	**Anthropometrics Weight (kgs)** **Height (cms) BMI (Kg·m^**−2**^)**
**Cross-sectional studies**							
Bowyer et al., 1990 (18)	England	CHD	TGA post-surgery Mustard	Complex/repaired	20 (2F, 18M)	9 (6–12)^*^	Weight: 30 Height: 136
		Control	Healthy children		18 (3F, 15M)	9.5 (NR)	Weight: 31 Height: 137
Tomassoni et al., 1991 (33)	United States	CHD	TOF repaired	Complex/repaired	20 (9F, 11M)	9.93 ± 2.88 (SE)	Weight: 32.81 ± 12.35 (SE) Height: 136.07 ± 19.55 (SE)
		Control	Healthy children		20 (9 F, 11M)	10.22 ± 2.48 (SE)	Weight: 35.75 ± 11.47 (SE) Height: 139.98 ± 13.52 (SE)
Balderston et al., 1992 (34)	United States	CHD	CoA repaired	Complex/repaired	31 (9F, 22M)	11.2 (7–17)^*^	NR
		Control	Healthy children		22 (NR)	NR (7–17)^*^	NR
Takagi et al., 1994 (37)	Japan	CHD	CHD acyanotic and cyanotic post-surgery	Complex/simple/ repaired	Acyanotics: 15 Cyanotics: 21	Acyanotics: 10.7 ± 2.7 Cyanotics: 11.4 ± 3.9	NR
		Control	Healthy children		16 (NR)	10.9 ± 2.7	NR
Douard et al., 1997 (19)	France	CHD	TGA post Senning surgery	Complex/repaired	43 (11F, 32M)	12 ± 3.1	Weight: 38.2 ± 15.9 Height: 145 ± 17
		Control	Healthy children		43 (NR)	12.8 ± 3.4	Weight: 44.6 ± 15 Height: 154 ± 19
Buheitel et al., 2000 (25)	Germany	CHD	CHD complex post Fontan and TGA post Senning	Complex/repaired	Fontan: 21 (11F, 10M) TGA: 13 (8F, 5M)	Fontan: 11.1 ± 2.5 TGA: 11.8 ± 1.9	Fontan: Weight: 34.2 ± 10.3 Height: 144 ± 14 TGA: Weight: 38.0 ± 11.6 Height: 148 ± 12
		Control	Healthy children		21 (11F, 10M)	11.2 ± 2.3	Weight: 36.0 ± 10.9 Height: 143 ± 13
Pfammatter et al., 2002 (26)	Switzerland	CHD	ASD repaired	Simple/repaired	14 (9F, 5M)	11.4 (6.8-16.1)^**^	Weight: 40 (27–70)^**^
		Control	Healthy children		15 (9F, 6 M)	11 (7.8–15.8)^**^	Weight: 37 (28–53)^**^
Zajac et al., 2002 (27)	Poland	CHD	CHD post Fontan	Complex/repaired	14 (8F, 6 M)	8.1 (5.7–17)	NR
		Control	Healthy children		12 (6F, 6 M)	7.1 (6.1–16.8)^*^	NR
Norozi et al., 2005 (28)	Germany	CHD	TOF, PA, CoA, ASD/VSD	Simple/complex/ repaired	84 (40F, 47 M)	TOF: 8.2 ± 2.0 PA: 8.0 ± 2.5 CoA: 7.9 ± 2.2 ASD/VSD: 7.7 ± 1.7	TOF: Weight: 27.7 ± 9.4 Height: 130 ± 14 PA: Weight: 27.8 ± 9.5 Height: 130 ± 15 CoA: Weight: 26.1 ± 7 Height: 127 ± 13 ASV/VSD: Weight: 25 ± 5 Height: 127 ± 10
		Control	Healthy children		98 (49F, 49M)	7.8 ± 1.8	Weight: 27.3 ± 5.7 Height: 129 ± 11
Binkhorst et al., 2008 (29)	Netherlands	CHD	VSD repaired and VSD with conservative treatment	Simple/repaired/not repaired	27 (14F, 13M)	VSD repaired: 13 ± 2.5 VSD conservative: 12.5 ± 3	VSD repaired: Weight: 49 ± 15 Height: 158 ± 16 BMI: 19 ± 3 VSD conservative: Weight: 51 ± 18 Height: 159 ± 13 BMI: 20 ± 5
		Control	Healthy children		15 (8F, 7M)	12.5 ± 3	Weight: 49 ± 15 Height: 159 ± 17 BMI: 19 ± 2.5
Moalla et al., 2008 (30)	France	CHD	Complex CHDs repaired in NYHA Class II or III	Complex/repaired	12 (NR)	13.0 ± 1.2	Weight: 48.8 ± 5.2 Height: 159.4 ± 4.6 BMI 19.2 ± 1.9
		Control	Healthy children		12 (NR)	12.9 ± 1.1	Weight: 49.0 ± 10.2 Height: 156.5 ± 9.3 BMI: 19.9 ± 3.3
Van Beek et al., 2010 (31)	Netherlands	CHD	TGA post Arterial Switch	Complex/repaired	17 (5F, 12M)	12.1 ± 2.0	Weight: 47.3 ± 14.1 Height: 156.2 ± 14.6 BMI: 19.1 ± 2.4
		Control	Healthy children		20 (7F, 13M)	12.8 ± 2.4	Weight: 49.5 ± 10.6 Height: 159.9 ± 12.2 BMI: 19.2 ± 2.3
Kotby et al., 2012 (38)	Egypt	CHD	TOF repaired	Complex/repaired	21 (5F, 16M)	8 (5-13)	NR
		Control	Healthy children		15 (NR)	8 (5-13)	NR
Müller et al., 2013 (32)	Germany	CHD	CHD simple, moderate, and complex. NYHA I or II	Simple/complex/ repaired/no repaired	88 (36F, 52M)	12.7 (12-13.3)	BMI:18.5 (16.7–21.6)^***^
		Control	Healthy children		88 (36F, 52M)	12.5 (12.1-13.1)	BMI: 18.9 (17.1–21.8)^***^
Mazurek et al., 2016 (20)	Poland	CHD	TOF repaired, TGA repaired, CHD post Fontan NYHA I	Complex/repaired	42 (NR)	14 ± 2.72	NR
		Control	Healthy children		20 (NR)	14.90 ± 2.48	NR
Samos et al., 2016 (36)	Brazil	CHD	TGA post arterial switch	Complex/repaired	31 (12F, 19M)	10.2 ± 5.2	Weight: 17.0 ± 2.8 Height: 136 ± 17 BMI: 17.0 ± 2.8
		Control	Healthy children		29 (8F, 21M)	10.9 ± 4.3	Weight: 18.1 ± 3.5 Height: 147 ± 14 BMI: 18.1 ± 3.5
Amedro et al., 2018 (21)	France	CHD	Simple, moderate and complex CHD	Simple/complex/ repaired/ no repaired	496 (NR)	12.2 ± 3.3	Weight: 44.1 ± 15.8 Height: 150.9 ± 17.5 BMI: 18.7 ± 3.6
		Control	Healthy children		302 (NR)	11.1 ± 2.6	Weight: 42.2 ± 13.3 Height: 150.0 ± 16.0 BMI: 18.3 ± 2.9
Hock et al., 2018 (22)	Germany	CHD	CHD post Fontan	Complex/repaired	41 (NR)	12.0 ± 3.2	Weight: 39.1 ± 14.5 Height: 145.2 ± 18.7
		Control	Healthy children		121 (NR)	12.6 ± 2.4	Weight: 47.0 ± 14.2 Height: 155.0 ± 13.6
Coomans et al., 2020 (35)	United States	CHD	TOF repaired	Complex/repaired	45 (14F, 31M)	13.9 ± 2.9	Weight: 50.6 ± 18.3 Height: 157.2 ± 15.0
		Control	Healthy children		45 (14F, 31M)	13.9 ± 2.8	Weight: 4.8 ± 16.3 Height: 159.6 ± 18.0
Gavotto et al., 2020 (23)	France	CHD	Simple, moderate and complex CHD	Simple/complex/ repaired/no repaired	407 (179F, 228M)	12.2 ± 3.4	Weight: 44.3 ± 15.9 Height: 150.9 ± 17.6
		Control	Healthy children		302 (130F, 172M)	11.1 ± 2.6	Weight: 42.2 ± 13.3 Height: 150.0 ± 16.0
**Controlled intervention studies**							
Moalla et al., 2005 (24)	France	CHD	Complex CHD repaired. NYHA class II or III.	Complex/repaired	17 (NR)	13.5 ± 0.5 (SE)	Weight: 50.5 ± 3.3 Height: 161.1 ± 1.5 BMI: 19.6 ± 1 (SE)
		Control	Healthy children		14 (NR)	12.9 ± 0.3 (SE)	Weight: 49.1 ± 2.8 Height: 57.0 ± 2.5 BMI: 20.0 ± 0.9 (SE)

*Data are shown as mean ± SD*,

* *mean range*,

** *median (range)*,

*** *median (interquartile range)*.

*ASD, atrial septal defect; BMI, body mass index; CHD, congenital heart disease; CoA, coarctation of the aorta; F, female; M, male; NR, not reported; NYHA, new york heart association; PA, pulmonary atresia; SE, standard error; TGA, transposition of the great arteries; TOF, tetralogy of fallot; VSD, ventricular septal defect*.

### Participants

In total, 1540 participants with CHD and 1248 healthy controls were enrolled in the included studies. Among the studies, the CHD sample size ranged from 12 (30) to 496 (21). Age varied from 7.7 ± 1.7 (28) to 14 ± 2.72 (20). The majority of the studies included only complex CHD (18–20, 22, 25, 27, 31, 33–36, 38), and nine studies included both simple and complex CHD (21–24, 26, 28–30, 32, 37). Sixteen studies included only repaired CHD (18–20, 22, 24–27, 30, 31, 33–38); however, only ten studies reported the age of surgery, which was before 3 years old in the majority of the cases (19, 22, 26–28, 30, 33). A summary of patients' characteristics is presented in Table 1.

### Type of Assessment

To assess exercise capacity, 18 studies used the direct CPET method: seven of them performed the CPET on a treadmill (18–20, 27, 36–38) and 13 in a cycle ergometer (21–26, 29–35). One study used the 6MWT (24). The results of the studies that used the CPET to evaluate exercise capacity are shown in Table 2. The quantitative variables (VO_2_peak, Wmax, VE/CO_2_ slope, O_2_ Pulse, and HRmax) expressed as mean and SD were analyzed using a meta-analysis method. Results from Bowyer et al. (18) and Binkhorst et al. (29) were not included in the meta-analysis because they reported their results in other units.

**Table 2:** Results of the Cardiopulmonary Test of the included studies.

**Authors, year**	**Group**	**Test Protocol**	**VO_**2**_peak (ml·min^**−1**^ · kg^**−1**^)**	**VE/VCO_**2**_** **Slope**	**Maximum load (Wmax)**	**Pulse of O_**2**_** **(ml·beat^**−1**^)**	**HR máx (bpm)**
**Cross-sectional studies**							
Bowyer et al., 1990 (18)	CHD	Treadmill Bruce Protocol	38	NR	NR	NR	175
	Control		52	NR	NR	NR	195
Tomassoni et al., 1991 (33)	CHD	Cycloergometer Bruce and modified Bruce protocol in children under 8 years	34.10 ± 2.98 (SE)	NR	NR	NR	173.8 ± 4.6 (SE)
	Control		37.53 ± 2.45 (SE)	NR	NR	NR	184.5 ± 2.9 (SE)
Balderston et al., 1992 (34)	CHD	Cycloergometer James Protocol	48.1 ± 1.4	NR	73 ± 4	NR	183 ± 21
	Control		49 ± 2.1	NR	78 ± 6	NR	189 ± 3
Takagi et al., 1994 (37)	CHD	Treadmill Bruce protocol	Acyanotics: 48.5 ± 11.0 Cyanotics: 36.1 ± 9.9	NR	NR	NR	Acyanotics: 183.4 ± 16.1 Cyanotics: 178.1 ± 16.0
	Control		52.7 ± 8.9	NR	NR	NR	189.8 ± 9.1
Douard et al., 1997 (19)	CHD	Treadmill Bruce protocol	32.6 ± 5.6	36.9 ± 1.5	NR	7.4 ± 2.9	166 ± 20
	Control		44.7 ± 6.1	31.4 ± 5.3	NR	10.7 ± 4.2	188 ± 16
Buheitel et al., 2000 (25)	CHD	Cycloergometer Ramp protocol	Fontan: 36.5 ± 5.7 Senning: 37.5 ± 7.1	NR	W/Kg Fontan: 2.0 ± 0.4 Senning: 2.2 ± 0.4	Fontan: 239 ± 48 Senning: 251 ±78 (by Kg)	Fontan: 156 ± 22 Senning: 155 ±27 (by Kg)
	Control		44.6 ± 6.0	NR	W/Kg 2.6 ± 0.4	O_2_ pulse/ kg: 275 ± 49	165 ± 22
Pfammatter et al., 2002 (26)	CHD	Cycloergometer	37.8 (28.5-48.6)	NR	W/kg 3.2 (1.8-4.0)^**^	NR	180 (142-191)^**^
	Control		44.3 (30.9-52.3)	NR	W/kg 2.9 (2.0–4.0)^**^	NR	191 (152-202)^**^
Zajac et al., 2002 (27)	CHD	Treadmill Modified bruce protocol	14.4 ± 6.1	NR	80.8 ± 45.7	2.57 ± 1.23	142.2 ± 24.8
	Control		30.9 ± 7.6	NR	238.4 ± 63.5	6.14 ± 2.23	183.4 ± 23.6
Binkhorst et al., 2008 (29)	CHD	Cycloergometer Ramp protocol	VSD repaired: 45 ± 9 VSD Conservative: 46 ± 7	NR	VSD repaired: 3.4 ± 0.7 VSD Conservative: 3.4 ± 0.6 (W/Kg)	NR	VSD repaired: 179 ± 8 VSD Conservative: 188 ± 6
	Control		48 ± 8	NR	3.7 ± 0.9	NR	188 ± 8
Moalla et al., 2008 (30)	CHD	Cycloergometer Ramp protocol	30.2 ± 6.1	NR	107 ± 17	NR	170 ± 17
	Control		46.5 ± 6.7	NR	159.6 ± 26.7	NR	197 ± 10
Van Beek et al., 2010 (31)	CHD	Cycloergometer Ramp protocol	41.1 ± 6.6	NR	154.1 ± 61.6	NR	180 ± 14
	Control		47.4 ± 6.4	NR	179.3 ± 60.5	NR	189 ± 9
Müller et al., 2013 (32)	CHD	Cycloergometer	35.5 (31.3-41.0)^***^	27.7 (25.4-29.8)^***^	117 (94-133)^***^	ml/kg: 0.20 (0.18-0.24)^***^	175 (161-184)^***^
	Control		42.4 (36.1-47.3)^***^	27.8 (25.7-29.9)^***^	159 (143-193)^***^	ml/kg: 0.22 (0.19-0.26)^***^	187 (181-196)^***^
Mazurek et al., 2016 (20)	CHD	Treadmill Ramp protocol	34.6 ± 8.0*	NR	NR	NR	NR
	Control		38.4 ± 7.7	NR	NR	NR	NR
Samos et al., 2016 (36)	CHD	Treadmill Ramp protocol	40.52 ± 7.19	35.73 ± 4.94	NR	7.83 ± 2.8	162.97 ± 17.88
	Control		45.47 ± 8.05	34.75 ± 5.39	NR	9.68 ± 4.50	201 ± 78.32
Amedro et al., 2018 (21)	CHD	Cycloergometer Ramp protocol	38.1 ± 8.1	NR	105.2 ± 71.6	NR	174.7 ± 18.8
	Control		43.5 ± 7.5	NR	111.5 ± 73.9	NR	187.5 ± 11.1
Hock et al., 2018 (22)	CHD	Cycloergometer Ramp protocol	34.8 ± 7.5	31.6 ± 3.3	125.2 ± 45.2	NR	NR
	Control		42.1 ± 8.4	27.5 ± 2.9	165.7 ± 41.3	NR	NR
Coomans et al., 2020 (35)	CHD	Cycloergometer Ramp protocol	34.46 ± 8.14	27.37 ± 3.88	112.2 ± 42.4	NR	174.0 ± 13.8
	Control		42.77 ± 8.14	25.09 ± 2.88	149.9 ± 65.7	NR	191.8 ± 9.4
Gavotto et al., 2020 (23)	CHD	Cycloergometer Ramp protocol	37.7 ± 6.9	NR	89.9 ± 44.3	NR	175.3 ± 15.8
	Control		42.6 ± 6.9	NR	121.8 ± 44.2	NR	187.6 ± 15.9
**Controlled intervention studies**							
Moalla et al., 2005 (24)	CHD	Cycloergometer Ramp protocol	28.9 ± 1.7 (SE)	NR	105.5 ± 5.8 (SE)	NR	163.5 ± 6 (SE)
	Control		46.5 ± 1.8 (SE)	NR	159.6 ± 7.1 (SE)	NR	197.2 ± 2.9 (SE)

*Data are shown as mean ± SD*,

** *median (range)*,

*** *median (interquartile range)*.

*CHD, congenital heart disease; NR, not reported; SE, standard error; VSD, ventricular septal defect*.

### Risk of Bias Assessment

Of the selected cross-sectional articles (n = 20), all were rated as “fair” (50–75% affirmative answers). The RCT study (*n* = 1) obtained a “fair” rating. The quality assessment results for the individual studies obtained using the NHBLI quality assessment tool are presented in the Supplementary File 1.

### Main Findings

#### Peak Oxygen Consumption

**Figure 2:**
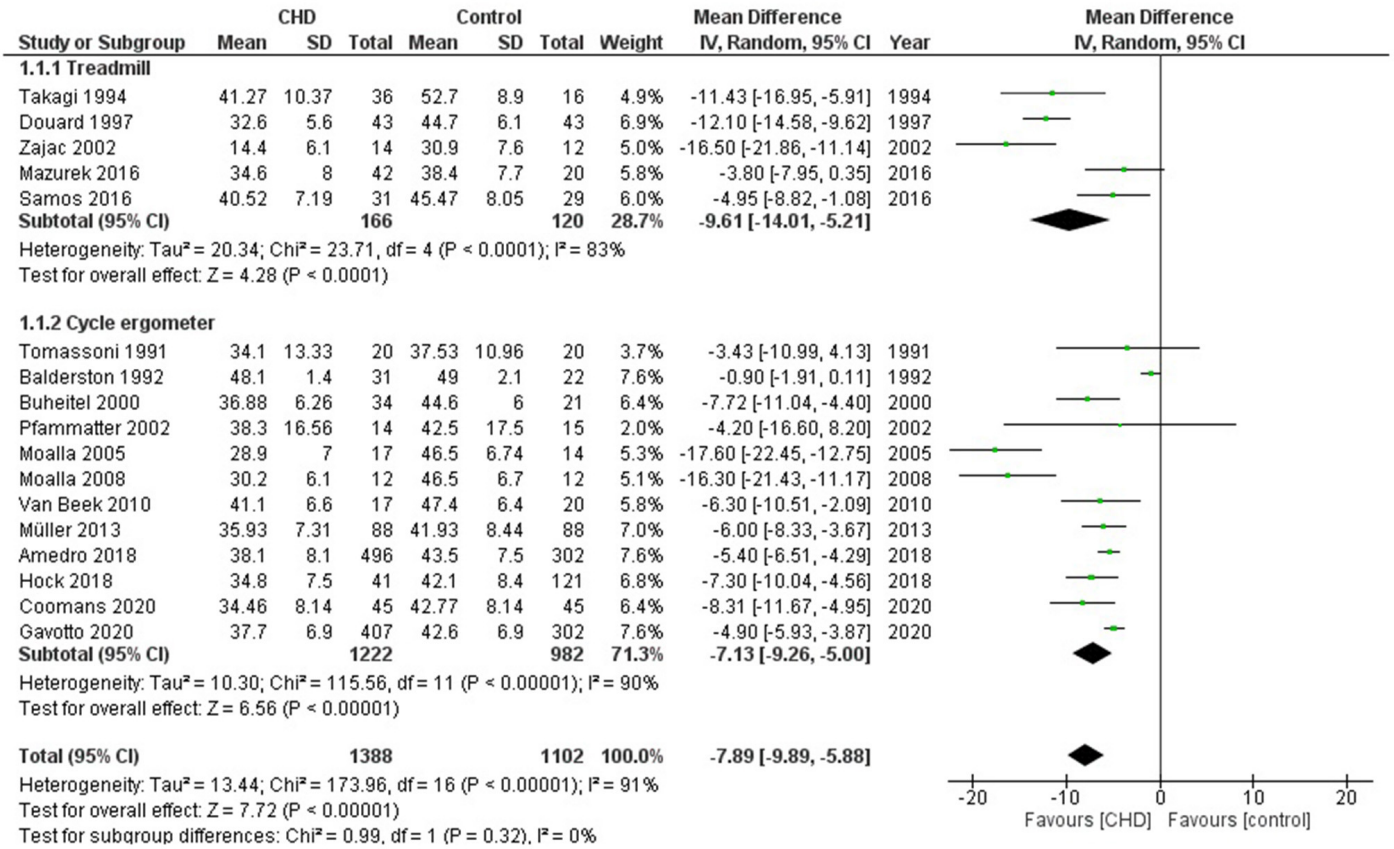
Forest plot for mean peak oxygen consumption for CHD in children and adolescents and healthy controls.

**Figure 3:**
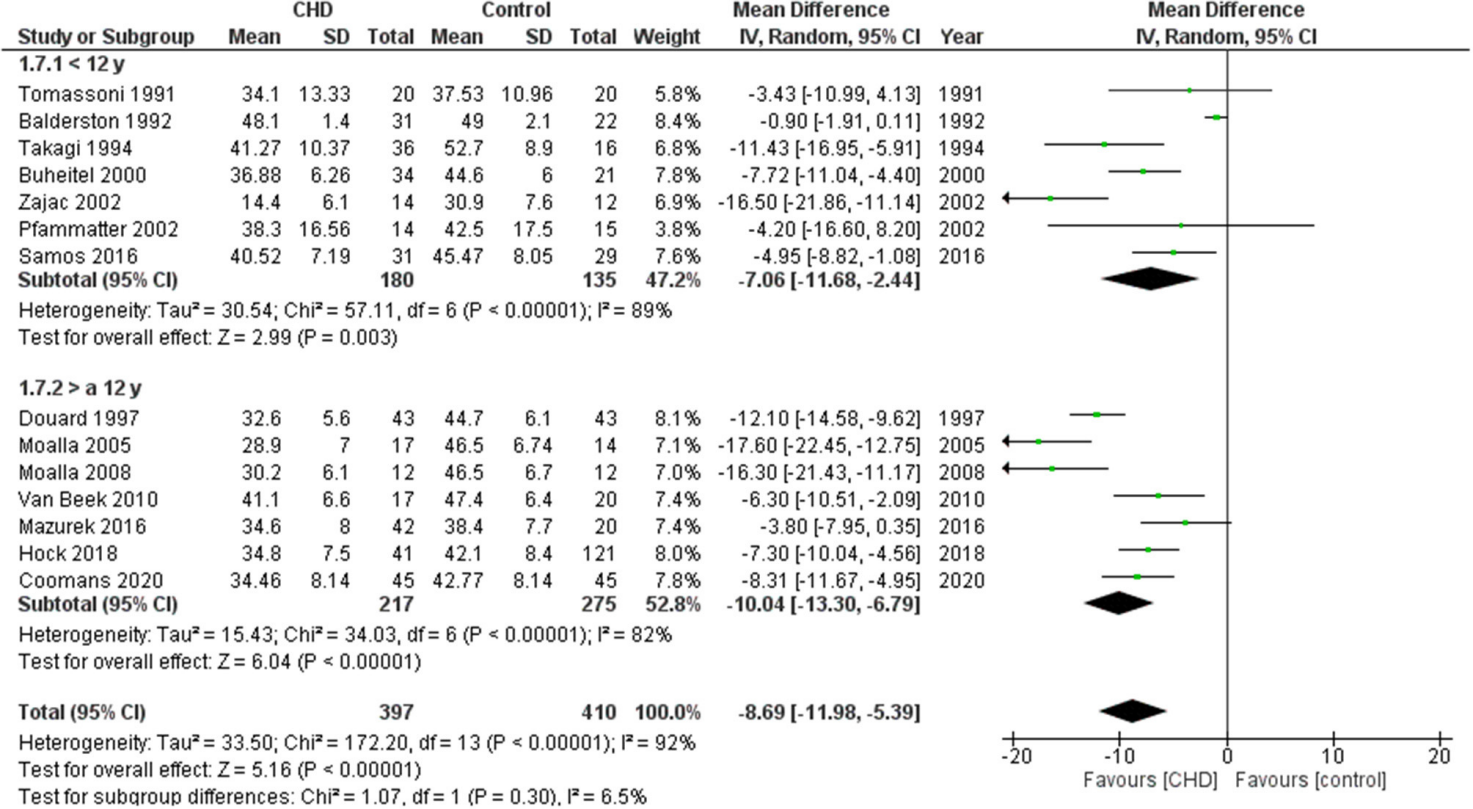
Forest plot for mean peak oxygen consumption for CHD in children and adolescents and healthy controls. Subanalysis by age.

Seventeen studies examined exercise capacity considering VO_2_peak (19–27, 30–37). These studies compared 1388 participants with CHD vs. 1102 healthy controls. The heterogeneity of the comparison was high (I^2^ = 91%). Participants with CHD averaged −7.9 ml/Kg/min (95% CI: −9.9, −5.9) of VO_2_peak compared with controls (*p* < 0.00001) (Figure 2). When considering the analysis by subgroups according to the type of test, those who performed the test on treadmill included 166 subjects with CHD and 120 controls, while on cycle ergometer it was 1222 CHD and 982 controls. Those who performed CPET on a treadmill had on average −9.6 ml/Kg/min (95% CI: −14.0, −5.2) of VO_2_peak compared with the control group (*p* < 0.0001) and those who performed test on cycle ergometer had an average of −7.1 ml/Kg/min (95% CI: −9.3, −5.0) of VO_2_peak as compared with the control group (p<0.00001). If we analyze only studies with patients surgically repaired (19, 20, 22, 24–27, 30, 31, 33–37), the participants with CHD averaged −8.7 ml/Kg/min (95% CI: −12.0, −5.4) of VO_2_peak compared with controls (*p* < 0.00001, I^2^ = 92%) (Supplementary File 2, Figure 1). In addition, we sub-analyzed by age range and observed that the VO_2_peak of the group under 12 years old had a decrease of −7.1 ml/Kg/min (95% CI: −11.7, −2.4) compared with the control group (*p* < 0.00001, I^2^ = 89%), and the group ≥ 12 years old had a reduction of −10.0 ml/Kg/min (95% CI: −12.0, −5.3) compared with the control group (*p* < 0.00001, I^2^ = 82%) (Figure 3).

#### Maximum Workload

**Figure 4:**
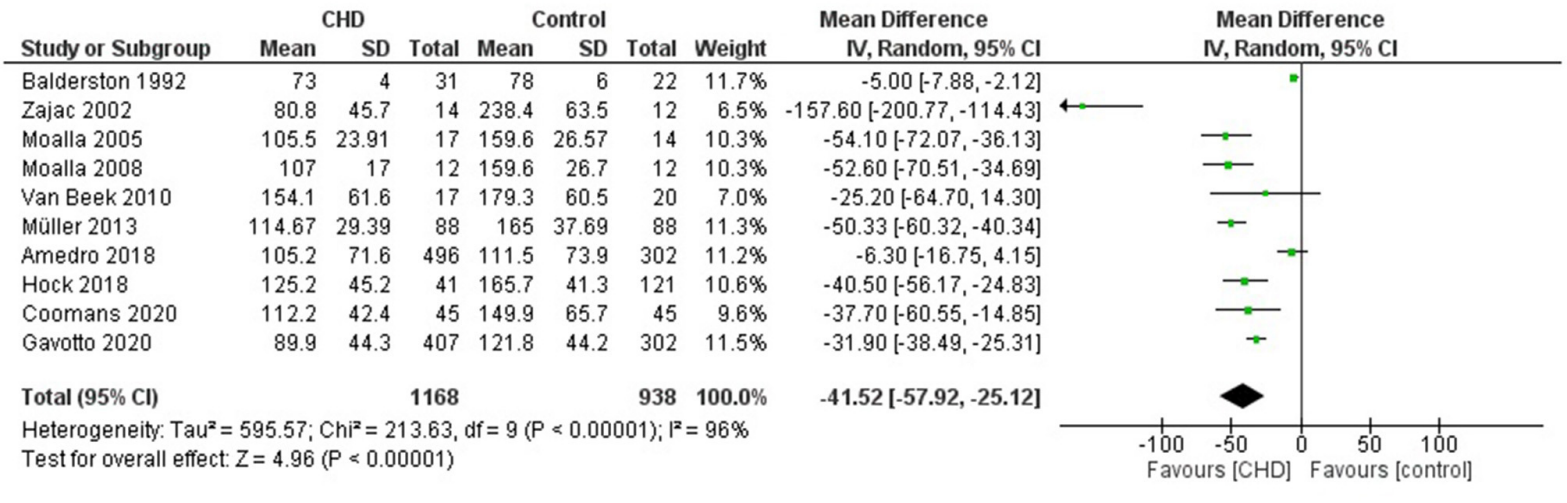
Forest plot for mean maximum workload for CHD in children and adolescents and healthy controls.

**Figure 5:**
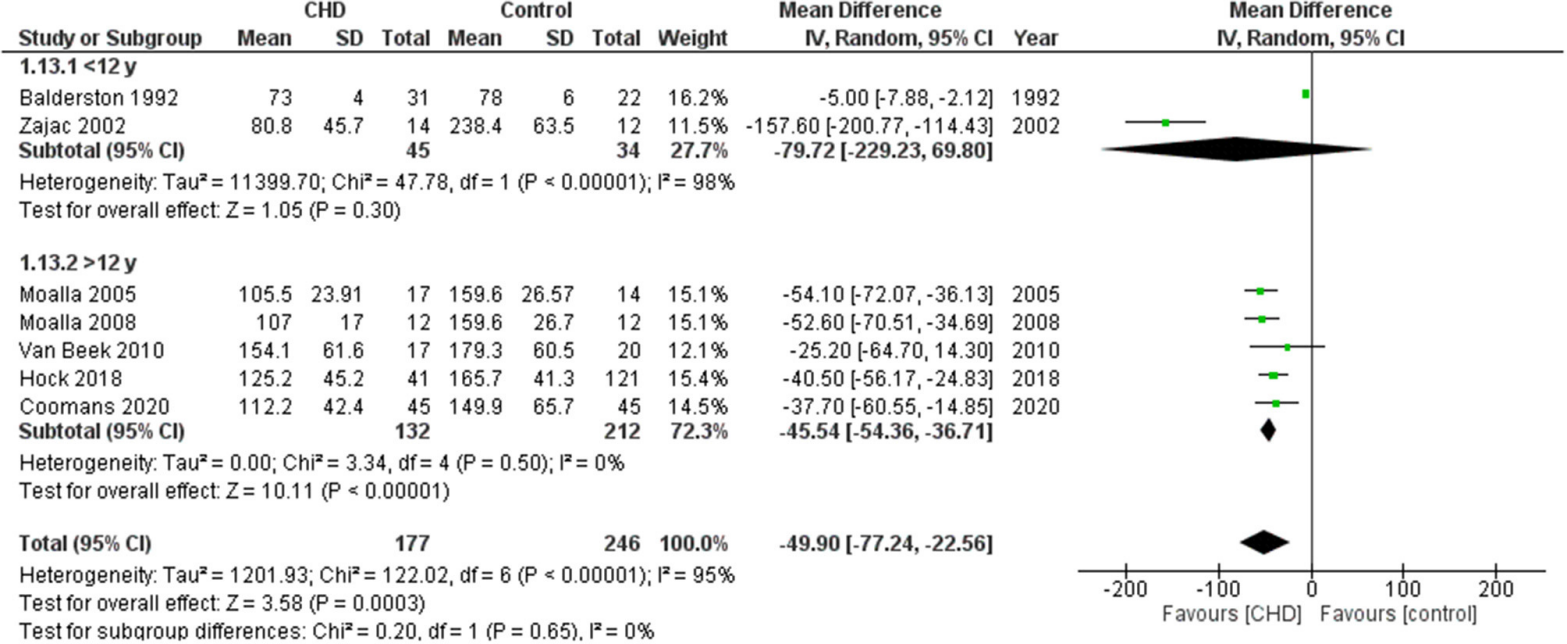
Forest plot for mean maximum workload for CHD in children and adolescents and healthy controls. Subanalysis by age.

Ten studies reported the Wmax in watts (21–24, 27, 30–32, 34, 35). These studies compared 1168 participants with CHD vs. 938 healthy controls. The heterogeneity of the comparison was high (I^2^ = 96%). Participants with CHD averaged −41.5 Wmax (95% CI: −57.9, −25.1 watts) compared with controls (*p* < 0.00001) (Figure 4). If we analyze only studies with patients surgically repaired (22, 24, 27, 30, 31, 34, 35) the participants with CHD averaged −49.9 watts (95% CI: −77.2, −22.6) of Wmax compared with controls (*p* < 0.0003, I^2^ = 95%) (Supplementary File 2, Figure 2). In addition, we sub-analyzed by age range and observed that the Wmax of the group under 12 years old was similar with the control group [−79.7 watts (95% CI: −229.2, 69.8), *p* = 0.3, I^2^ = 98%], and the group ≥ 12 years old had a reduction of −45.5 watts (95% CI: −54.4, −36.7) compared to the control group (*p* < 0.00001, I^2^ = 0%) (Figure 5).

#### VE/VCO_2_ Slope

**Figure 6:**
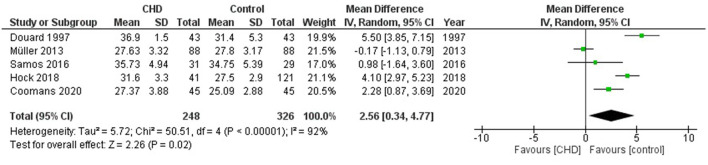
Forest plot for mean ventilatory equivalent for carbon dioxide at anaerobic threshold for CHD in children and adolescents and healthy controls.

Five studies examined the VE/VCO_2_ slope (19, 22, 32, 35, 36). These studies compared 248 participants with CHD vs. 326 healthy controls. The heterogeneity of the comparison was high (I^2^ = 92%). Participants with CHD had on average 2.6 more VE/VCO_2_ slope (95% CI: 0.3, 4.8) compared with controls (*p* < 0.02) (Figure 6). If we analyse only studies with patients surgically repaired (19, 22, 35, 36) the participants with CHD averaged 3.4 (95% CI: 1.7, 5.1) of VE/VCO_2_ compared with controls (*p* < 0.0001, I^2^ = 77%) (Supplementary File 2, Figure 3).

#### Oxygen Pulse

**Figure 7:**
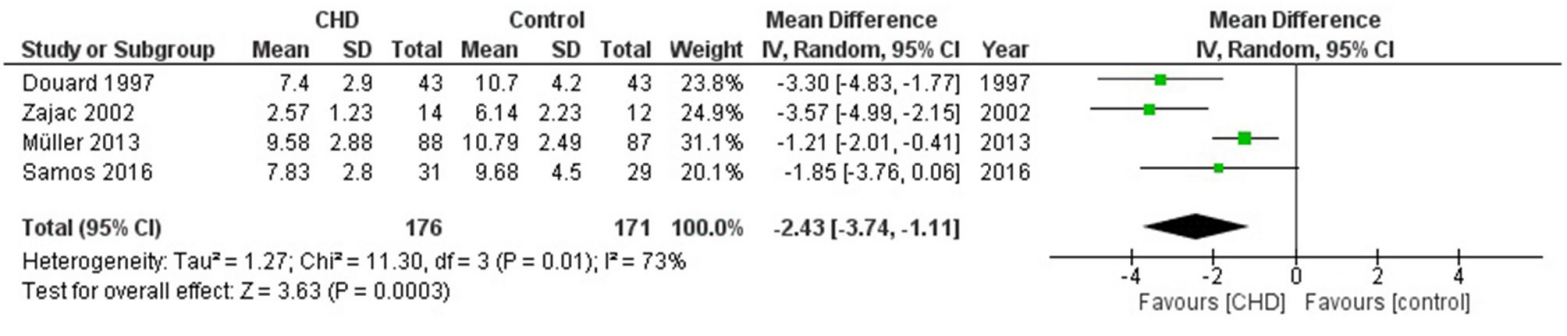
Forest plot for mean oxygen pulse for CHD in children and adolescents and healthy controls.

Four studies examined O_2_ pulse in ml/beat (19, 27, 32, 36). These studies compared 176 participants with CHD vs 171 healthy controls. The heterogeneity of the comparison was high (I^2^ = 73%). Participants with CHD had on average −2.4 ml/beat of O_2_ pulse (95% CI: −3.7, −1.1 ml/beat) compared with controls (*p* < 0.0003) (Figure 7). If we analyse only studies with patients surgically repaired (19, 27, 36) the participants with CHD averaged−3.1 ml/beat (95% CI: −4.0, −2.1) of O_2_ pulse compared with controls (*p* < 0.00001, I^2^ = 6%) (Supplementary File 2, Figure 4).

#### Maximum Heart Rate

**Figure 8:**
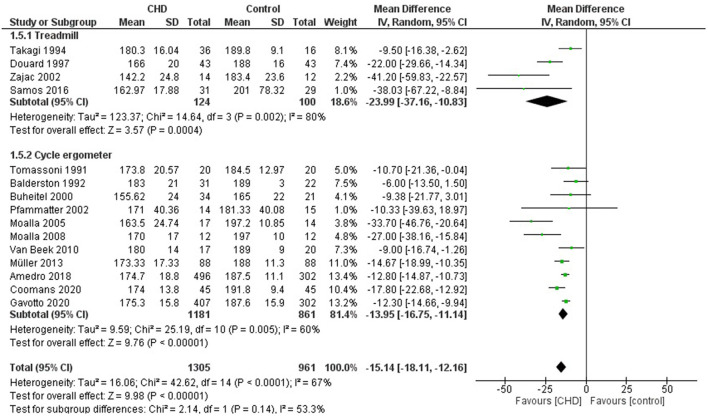
Forest plot for maximum heart rate for CHD in children and adolescents and healthy controls.

**Figure 9:**
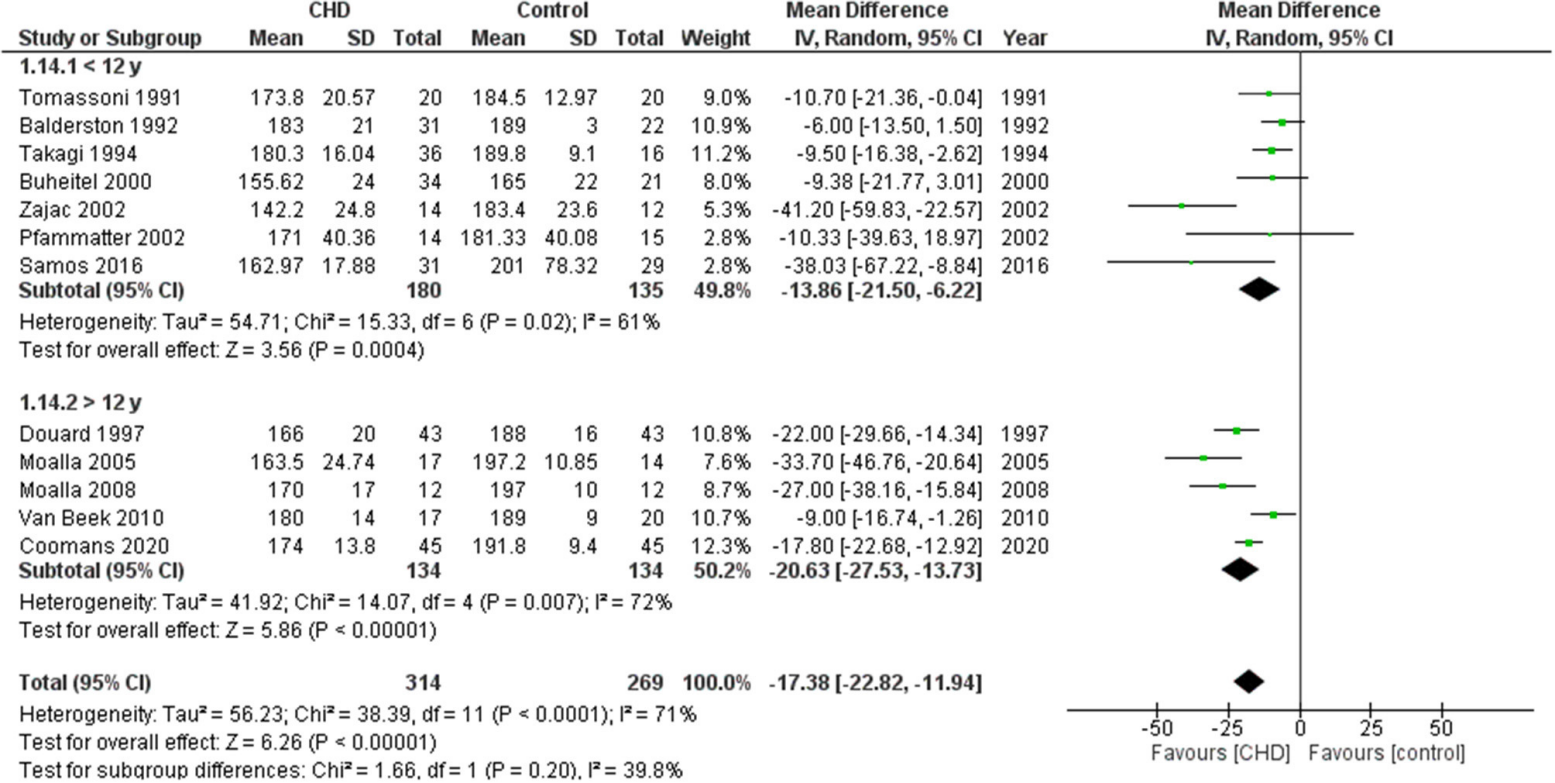
Forest plot for maximum heart rate for CHD in children and adolescents and healthy controls. Subanalysis by age.

Fifteen studies reported working HRmax in beats per minute (bpm) during CPET (19, 21, 23–27, 30–37). These studies compared 1305 participants with CHD vs. 961 healthy controls. The heterogeneity of the comparison was high (I^2^ = 67%). Participants with CHD averaged −15 bpm (95% CI: −18, −12 bpm) of HRmax compared with controls (*p* < 0.00001) (Figure 8). When considering the analysis by subgroups according to the test type, those who performed the test on a treadmill included 124 subjects with CHD and 100 controls, while on the cycle ergometer it was 1181 CHD and 861 controls. Those who performed the test on a treadmill had an average of −24 bpm (95% CI: −37, −11) of HRmax compared with the control group; in contrast, those who performed the test on a cycle ergometer had an average of −14 bpm (95% CI: −17, −11) of HRmax compared with the control group (*p* < 0.00001). If we analyze only studies with patients surgically repaired (19, 24–27, 30, 31, 33–37) the participants with CHD averaged −17 bpm (95% CI: −23, −12) of HRmax compared with controls (*p* < 0.0001, I^2^ = 71%) (Supplementary File 2, Figure 5). In addition, we sub-analyzed by age range and observed that the HRmax of the group under 12 years old had a decrease of −14 bpm (95% CI: −22, −6) compared with the control group (*p* = 0.0004, I^2^ = 61%), and the group ≥ 12 years old had a reduction of −21 bpm (95% CI: −28, −14) compared with the control group (*p* < 0.00001, I^2^ = 82%) (Figure 9).

## Discussion

This systematic review with meta-analysis of observational studies showed that children and adolescents with CHD have a significant decrease in the exercise capacity compared with healthy controls of similar age. VO_2_peak, HRmax, Wmax, and O_2_ Pulse were significantly lower in children and adolescents with partially or fully repaired CHD compared with healthy controls.

Our results showed that children and adolescents with CHD have a reduction close to 8 ml/Kg/min, although 76% of the selected articles exclusively evaluated patients with repaired CHD, which shows a considerable residual effect of CHD. The significant impairment of VO_2_peak directly impacts on exercise capacity, morbidity and mortality and is a prognostic factor in this population, reaching clinical and functional relevance. The presence of pulmonary hypertension (PH) is also a condition that could affect exercise capacity evaluated through VO_2_peak; however, it was not reported in most articles. On the other hand, one of the few manuscripts which reported it did find that the presence of PH was significantly associated with lower VO_2_peak (21).

Coomans et al. (35) highlighted that, in patients with repaired Tetralogy of Fallot (TOF), VO_2_peak and HRmax is lower as compared with controls, attributing, in part, the lower performance to chronotropic insufficiency due to a positive and significant correlation between HRmax and VO_2_peak (*r* = 0.418; *p* < 0.01). Additionally, our results showed that patients with CHD, in the included studies, have a diminution close to 15 bpm compared with healthy peers. The decrease in HRmax is almost double in those who perform the test on a treadmill, which is in line with a greater decrease in VO_2_peak in this evaluation device. These results highlight the importance of chronotropic insufficiency in maximum exercise performance, limiting the physical performance of individuals with even repaired TOF and being in line with other publications with similar results (35).

The lower HRmax in subjects with CHD was also related to factors affecting the correct function of the sympathetic and parasympathetic nervous system, among which is ischemia and / or denervation resulting from various cardiac surgical procedures or, in cases of cyanotic CHD, due to chronic hypoxemia (39).

Since most of the articles investigated exercise capacity in subjects with repaired heart disease, we decided to perform a subanalysis with only this group. Surprisingly, the surgically repaired subjects had lower VO_2_peak, Wmax, O_2_ pulse, and HRmax and higher VE/VCO_2_. Several factors could influence this result.. On one hand, there could be a selection bias of the investigated subjects since the follow-up is stricter in those subjects who are more seriously sick. On the other hand, participants with surgical correction were more severe than those without surgery (23).

We also analyzed the influence of age on the reduced exercise capacity. Considering adolescents those subjects older than 12 years old, we showed a greater decrease in VO_2_peak and HRmax compared with children under 12. A special case was Wmax, which was shown to be reduced only in the group older than 12 years. These results lead us to think that the differences increase over the years and are more pronunced during adolescence. Although our research does not include adult patients, there are already reports with decreased exercise capacity similar or even greater than what we found in those over 12 years old (40, 41).

Subjects with incomplete CHD repair have significant reductions in age-adjusted peak work rates and peak ventilation compared with their counterparts who had complete repair surgery (42). Amedro et al. observed that, in 496 children with CHD who underwent CPET compared with controls, VO_2_peak alteration was more prevalent in most subjects with partial repair or complex CHD (single ventricles and complex anomalies of atrioventricular connections) (21). The lower VO_2_peak in their study group was also associated with right ventricular systolic hypertension and tricuspid regurgitation, which are frequently common in many right heart complex CHD cases. The literature has also highlighted this situation, especially in patients with TOF, transposition of the great arteries, and univentricular heart with Fontan physiology (43).

Sequential CPET studies in young adult subjects with Fontan physiology have emphasized the prognostic value of this test regarding survival, mortality and the need for transplantation. There is often a decrease in VO_2_peak that precedes these events (44). Cooney et al. postulate that a change in VO_2_peak is an independent prognostic factor, which may allow early identification of subjects who could benefit from more intensive and preventive management (44). The change in VO_2_peak between sequential CPETs predicts transplant-free survival during and above any risk predicted by a single VO_2_peak measurement. Studies that have followed individuals with Fontan physiology from childhood to adulthood have documented a gradual decline in VO_2_peak over the years (14, 45). The decrease in VO_2_peak with age may be a factor to be identified and considered in subjects with CHD as a selection parameter for timely cardiac rehabilitation programmes starting even in the early stages of life (schooling).

The VE/VCO_2_ slope is elevated in most subjects with heart failure as it is inversely related to CO at peak exercise and to pulmonary perfusion, a situation that could also be experienced in subjects with CHD (46). In addition, the elevation of this slope is commonly observed in pulmonary vascular anomalies such as PH (47), which can also be experienced in patients with partially repaired CHD due to greater physical exertion.

A decrease in the O_2_ pulse during progressive exercise could indicate circulatory insufficiency or cardiovascular limitation (48). It is generally associated with the appearance of PH and impaired cardiac perfusion. In combination with a sudden decrease in the VO_2_ / Wmax ratio, it could indicate myocardial ischemia (49). In addition, a low O_2_ pulse is indicative of a reduced cardiac index (CI) or stroke volume, which implies a greater dependence on HR to increase CO (48, 50), a situation that may be common to certain CHD considered in the different subgroups that constitute the total of subjects with CHD of this revision.

Although our objective was to measure exercise capacity and not physical activity, it is important to note that some articles reported it (31, 32). This is important because a previous investigation of our group has shown that the moderate-to-vigorous physical activity of 46% of children with CHD is less than what is recommended by WHO (51). However, Van Beek et al. and Müller et al. found that exercise capacity is decreased in children with CHD, but physical activity showed no differences between groups (31, 32). Even in Müller's study, both groups performed more physical activity than recommended by clinical guidelines (32).

Finally, although the search period was long, our results consider only studies from the last three decades that explored exercise capacity in a large cohort of children and adolescents with CHD worldwide, which gives high value to our main message.

### Limitations

Our study has some limitations. Most of the studies included subjects with different types of CHD, which may limit the extrapolation of the results and recommendations to the entire spectrum of children and adolescents with CHD; even though our results were statistically significant. The heterogeneous nature of CHD implies that many lesions have different pathophysiological behaviors and conditions, a wide spectrum of severity, as well as the implication or impact that suffering from associated comorbidities may influence the congenital health condition they may have. Another important limitation is that most studies do not report whether patients presented PH. This point is key since it is well known that PH determines lower exercise capacity. In this context, it is important to systematically review associations in order to establish in more detail the factors that can condition a certain result.

Additionally, there was heterogeneity in the control participants since some of them performed the CPET to get the authorization to play sports (22) and others who presented any symptoms although the CPET showed no disease (21, 23, 35). On the other hand, physical activity analysis was not included, which could have provided us information on how sedentary the population was, and were not evaluated respiratory exchange ratio (RER) that indirectly shows the muscle's oxidative capacity to get energy. Sedentarism, exercise and physically active lifestyles modify it.

Concerning the possible bias occurred in VO_2_peak and other CPET variables due to variability in different countries (with different daily levels of physical activity, for example, due to different cultural habits) it is important to remark that the comparisons of these parameters, both for the studies and the meta-analysis, were performed comparing patients to controls (adjusted for sex and age in each article) and not regarding their percentages of the predicted values. Therefore, using a control population was essential to avoid these biases.

## Conclusion

In conclusion, suffering from CHD in childhood and adolescence is associated with a lower exercise capacity as shown by worse VO_2_peak, Wmax, VE/VCO_2_ slope, O_2_ pulse, HRmax compared with healthy controls, not only in CPET but also in other variables as shown in indirect exercise capacity tests. These findings highlight the importance of carrying out a continuous evaluation and early determination of the factors associated with a potential decrease in exercise capacity. In this way, it would be possible to intervene in time by planning rehabilitation programmes and promoting an active lifestyle, since a lower exercise capacity can lead to a greater risk of morbidity, mortality, and deterioration of functionality.

## Data Availability Statement

The original contributions presented in the study are included in the article/Supplementary Material, further inquiries can be directed to the corresponding author.

## Author Contributions

YV-R: conceptualization, formal analysis, methodology, reviewing procedure and data extraction, writing—original draft, and writing—review & editing. JV-M: conceptualization, formal analysis, methodology, writing—original draft, and writing—review & editing. RT-C: conceptualization, formal analysis, methodology, supervision, writing—original draft, and writing—review & editing. LV-C: reviewing procedure and data extraction, writing—original draft, and writing—review & editing. GM: supervision and writing—review & editing. JV and IB: writing —original draft and writing—review & editing. All authors contributed to the article and approved the submitted version.

## Funding

This study has been funded by Instituto de Salud Carlos III (ISCIII) through the projects (PI17/01515 and PI21/00555) and co-funded by the European Union. RT-C was funded by a grant from the National Agency for Research and Development (ANID)/Scholarship Program/DOCTORADO BECAS CHILE/2018-72190117.

## Conflict of Interest

The authors declare that the research was conducted in the absence of any commercial or financial relationships that could be construed as a potential conflict of interest.

## Publisher's Note

All claims expressed in this article are solely those of the authors and do not necessarily represent those of their affiliated organizations, or those of the publisher, the editors and the reviewers. Any product that may be evaluated in this article, or claim that may be made by its manufacturer, is not guaranteed or endorsed by the publisher.

## Abbreviations

6MWT: Six-minute walking test
CHD: Congenital heart disease
CI: Cardiac index
CO: Cardiac output
CPET: Cardiopulmonary exercise test
HRmax: Maximum Heart rate
NHLBI: National Heart, Lung and Blood Institute
PH: Pulmonary hypertension
PRISMA: Preferred reporting items for systematic reviews and meta-analyses
PROSPERO: International prospective register of systematic reviews
RCTs: Randomized clinical trials
SV: Stroke volume
SWT: Shuttle walking test
TUG: Timed up and go test
V_E_: Minute ventilation
V_E_/VCO_2_: Ventilatory equivalent for carbon dioxide
V_E_/VCO_2_ slope: Minute ventilation/carbon dioxide production slope
VO_2_: Oxygen consumption
VO_2_peak: Peak oxygen consumption
VO_2_max: Maximum oxygen consumption
Wmax: Maximum workload.
