# Supplementary material for: Exercise Capacity in Children and Adolescents With Congenital Heart Disease: A Systematic Review and Meta-Analysis
Source: Front Cardiovasc Med. 2022 May 4;9:874700. doi: 10.3389/fcvm.2022.874700 (PMC9114479; doi:10.3389/fcvm.2022.874700)

Supplementary file 2

Figure 1

Forest plot for mean peak oxygen consumption for CHD in children and adolescents and healthy controls. Only studies with patients surgically repaired.

	CHD			Control				Mean Difference	
Study or Subgroup	Mean	SD	Total	Mean	SD	Total	Weight	IV, Random, 95% CI	Year
Tomassoni 1991	34.1	13.33	20	37.53	10.96	20	5.8%	-3.43 [-10.99, 4.13]	1991
Balderston 1992	48.1	1.4	31	49	2.1	22	8.4%	-0.90 [-1.91, 0.11]	1992
Takagi 1994	41.27	10.37	36	52.7	8.9	16	6.8%	-11.43 [-16.95, -5.91]	1994
Douard 1997	32.6	5.6	43	44.7	6.1	43	8.1%	-12.10 [-14.58, -9.62]	1997
Buheitel 2000	36.88	6.26	34	44.6	6	21	7.8%	-7.72 [-11.04, -4.40]	2000
Zajac 2002	14.4	6.1	14	30.9	7.6	12	6.9%	-16.50 [-21.86, -11.14]	2002
Pfammatter 2002	38.3	16.56	14	42.5	17.5	15	3.8%	-4.20 [-16.60, 8.20]	2002
Moalla 2005	28.9	7	17	46.5	6.74	14	7.1%	-17.60 [-22.45, -12.75]	2005
Moalla 2008	30.2	6.1	12	46.5	6.7	12	7.0%	-16.30 [-21.43, -11.17]	2008
Van Beek 2010	41.1	6.6	17	47.4	6.4	20	7.4%	-6.30 [-10.51, -2.09]	2010
Mazurek 2016	34.6	8	42	38.4	7.7	20	7.4%	-3.80 [-7.95, 0.35]	2016
Samos 2016	40.52	7.19	31	45.47	8.05	29	7.6%	-4.95 [-8.82, -1.08]	2016
Hock 2018	34.8	7.5	41	42.1	8.4	121	8.0%	-7.30 [-10.04, -4.56]	2018
Coomans 2020	34.46	8.14	45	42.77	8.14	45	7.8%	-8.31 [-11.67, -4.95]	2020
**Total (95% CI)**			**397**			**410**	**100.0%**	**-8.69 [-11.98, -5.39]**	

Heterogeneity: $Tau^2 = 33.50$; $Chi^2 = 172.20$, df = 13 (P < 0.00001); $I^2 = 92\%$
Test for overall effect: Z = 5.16 (P < 0.00001)

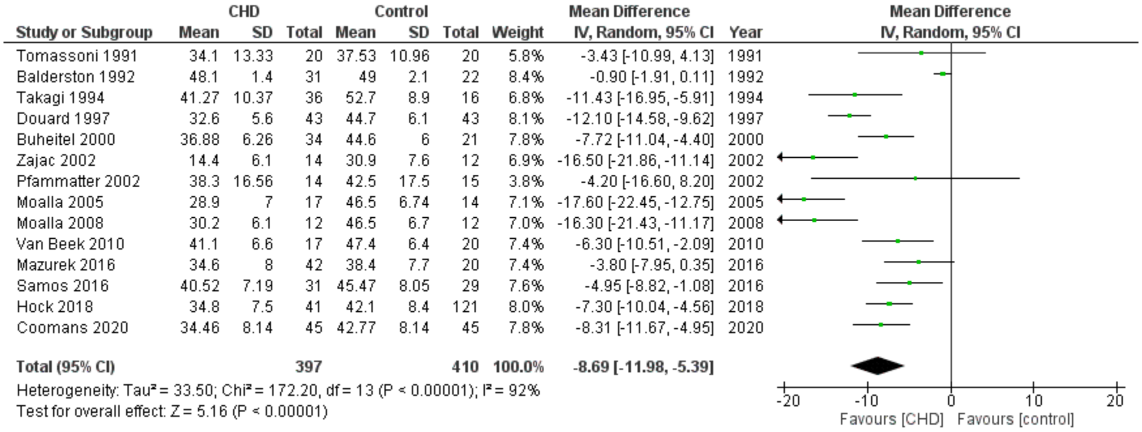

Figure 2

Forest plot for mean maximum workload for CHD in children and adolescents and healthy controls. Only studies with patients surgically repaired.

	CHD			Control				Mean Difference	
Study or Subgroup	Mean	SD	Total	Mean	SD	Total	Weight	IV, Random, 95% CI	Year
Balderston 1992	73	4	31	78	6	22	16.2%	-5.00 [-7.88, -2.12]	1992
Zajac 2002	80.8	45.7	14	238.4	63.5	12	11.5%	-157.60 [-200.77, -114.43]	2002
Moalla 2005	105.5	23.91	17	159.6	26.57	14	15.1%	-54.10 [-72.07, -36.13]	2005
Moalla 2008	107	17	12	159.6	26.7	12	15.1%	-52.60 [-70.51, -34.69]	2008
Van Beek 2010	154.1	61.6	17	179.3	60.5	20	12.1%	-25.20 [-64.70, 14.30]	2010
Hock 2018	125.2	45.2	41	165.7	41.3	121	15.4%	-40.50 [-56.17, -24.83]	2018
Coomans 2020	112.2	42.4	45	149.9	65.7	45	14.5%	-37.70 [-60.55, -14.85]	2020
**Total (95% CI)**			**177**			**246**	**100.0%**	**-49.90 [-77.24, -22.56]**	

Heterogeneity: $Tau^2 = 1201.93$; $Chi^2 = 122.02$, df = 6 (P < 0.00001); $I^2 = 95\%$
Test for overall effect: Z = 3.58 (P = 0.0003)

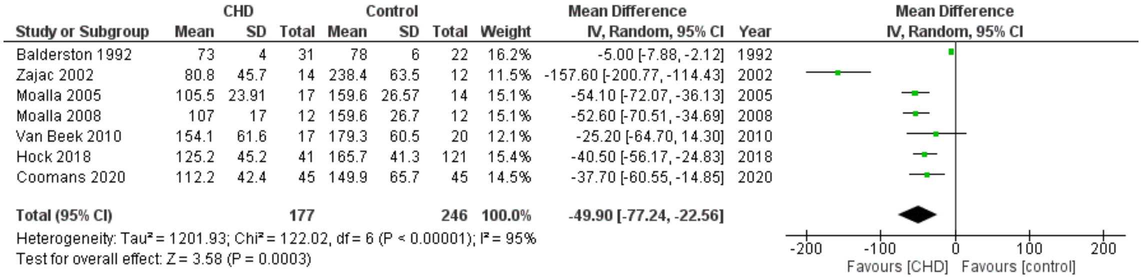

Figure 3

Forest plot for mean ventilatory equivalent for carbon dioxide at anaerobic threshold for CHD in children and adolescents and healthy controls. Only studies with patients surgically repaired.

	CHD			Control				Mean Difference	
**Study or Subgroup**	**Mean**	**SD**	**Total**	**Mean**	**SD**	**Total**	**Weight**	**IV, Random, 95% CI**	**Year**
Douard 1997	36.9	1.5	43	31.4	5.3	43	25.4%	5.50 [3.85, 7.15]	1997
Samos 2016	35.73	4.94	31	34.75	5.39	29	18.3%	0.98 [-1.64, 3.60]	2016
Hock 2018	31.6	3.3	41	27.5	2.9	121	29.2%	4.10 [2.97, 5.23]	2018
Coomans 2020	27.37	3.88	45	25.09	2.88	45	27.1%	2.28 [0.87, 3.69]	2020
**Total (95% CI)**			**160**			**238**	**100.0%**	**3.39 [1.73, 5.05]**	

Heterogeneity: Tau² = 2.13; Chi² = 13.05, df = 3 (P = 0.005); I² = 77%
Test for overall effect: Z = 4.00 (P < 0.0001)

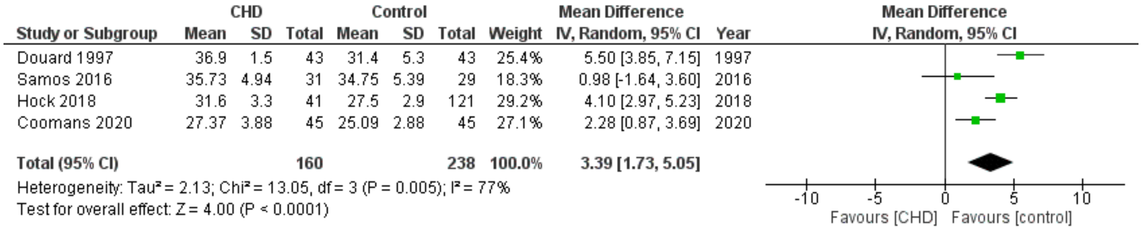

Figure 4

Forest plot for mean oxygen pulse for CHD in children and adolescents and healthy controls. Only studies with patients surgically repaired.

	CHD			Control				Mean Difference	
**Study or Subgroup**	**Mean**	**SD**	**Total**	**Mean**	**SD**	**Total**	**Weight**	**IV, Random, 95% CI**	**Year**
Douard 1997	7.4	2.9	43	10.7	4.2	43	35.7%	-3.30 [-4.83, -1.77]	1997
Zajac 2002	2.57	1.23	14	6.14	2.23	12	41.0%	-3.57 [-4.99, -2.15]	2002
Samos 2016	7.83	2.8	31	9.68	4.5	29	23.3%	-1.85 [-3.76, 0.06]	2016
**Total (95% CI)**			**88**			**84**	**100.0%**	**-3.07 [-4.02, -2.13]**	

Heterogeneity: Tau² = 0.04; Chi² = 2.13, df = 2 (P = 0.34); I² = 6%
Test for overall effect: Z = 6.38 (P < 0.00001)

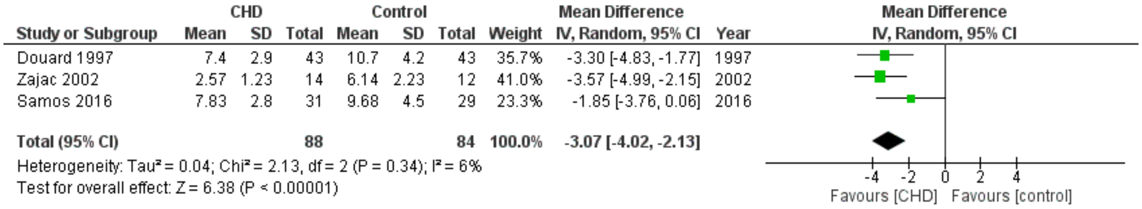

Figure 5

Forest plot for maximum heart rate for CHD in children and adolescents and healthy controls. Only studies with patients surgically repaired.

	CHD			Control				Mean Difference	
**Study or Subgroup**	**Mean**	**SD**	**Total**	**Mean**	**SD**	**Total**	**Weight**	**IV, Random, 95% CI**	**Year**
Tomassoni 1991	173.8	20.57	20	184.5	12.97	20	9.0%	-10.70 [-21.36, -0.04]	1991
Balderston 1992	183	21	31	189	3	22	10.9%	-6.00 [-13.50, 1.50]	1992
Takagi 1994	180.3	16.04	36	189.8	9.1	16	11.2%	-9.50 [-16.38, -2.62]	1994
Douard 1997	166	20	43	188	16	43	10.8%	-22.00 [-29.66, -14.34]	1997
Buheitel 2000	155.62	24	34	165	22	21	8.0%	-9.38 [-21.77, 3.01]	2000
Zajac 2002	142.2	24.8	14	183.4	23.6	12	5.3%	-41.20 [-59.83, -22.57]	2002
Pfammatter 2002	171	40.36	14	181.33	40.08	15	2.8%	-10.33 [-39.63, 18.97]	2002
Moalla 2005	163.5	24.74	17	197.2	10.85	14	7.6%	-33.70 [-46.76, -20.64]	2005
Moalla 2008	170	17	12	197	10	12	8.7%	-27.00 [-38.16, -15.84]	2008
Van Beek 2010	180	14	17	189	9	20	10.7%	-9.00 [-16.74, -1.26]	2010
Samos 2016	162.97	17.88	31	201	78.32	29	2.8%	-38.03 [-67.22, -8.84]	2016
Coomans 2020	174	13.8	45	191.8	9.4	45	12.3%	-17.80 [-22.68, -12.92]	2020
**Total (95% CI)**			**314**			**269**	**100.0%**	**-17.38 [-22.82, -11.94]**	

Heterogeneity: Tau² = 56.23; Chi² = 38.39, df = 11 (P < 0.0001); I² = 71%
Test for overall effect: Z = 6.26 (P < 0.00001)

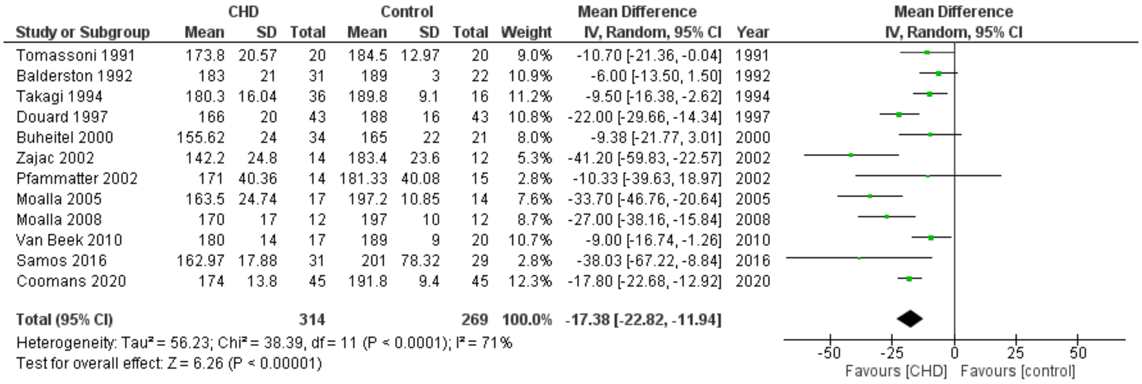

Supplement: Supplementary file 2 [file Data_Sheet_2.pdf]
